# Effect of bone marrow stem cell conditioned medium on amiodarone-induced submandibular salivary gland and kidney damage in male albino rats

**DOI:** 10.1186/s12903-025-07542-6

**Published:** 2026-01-10

**Authors:** Elham H. Ahmed, Mohammed Abdelrahim Abdeen, Eman Azmy Saad

**Affiliations:** 1 Oral Biology Department, Faculty of Dentistry, Mansoura University, Mansoura, Egypt; 2 Department of Supplementary Medical Science (Human Anatomy), Faculty of Dentistry, Sinai University, North Sinai, Egypt; 3 University Students Hospital, Mansoura University, Mansoura, Egypt

**Keywords:** Amiodarone, Stem Cells, Conditioned Media, Salivary glands, Kidney

## Abstract

**Aim:**

This study aimed to demonstrate the histological changes in the submandibular salivary glands (SMSGs) and kidneys induced by Amiodarone in albino rats, and to assess the potential therapeutic effects of bone marrow stem cells conditioned medium (BMSCs-CM).

**Methods:**

Thirty adult male Sprague Dawley Albino rats (180–230 g, aged 3–4 months, were arbitrarily divided into three equal groups. Group I (control): received a balanced diet and water for six days without medication. Group II (Amiodarone Group): was administered Amiodarone intraperitoneally at 50 mg/kg/day for six days. Group III (Conditioned media (CM) Group): received the same treatment as Group II, followed by intravenous injection of 1 ml BMSCs-CM for four consecutive days. At the end of the experiment, all rats were euthanized, and salivary gland and kidney samples were collected for histological analysis using hematoxylin and eosin (H&E), Masson's trichrome, and caspase 3 staining. ELISA was used to quantify tissue levels of malondialdehyde (MDA) and catalase (CAT).

**Results:**

Histologically, Group III's findings were almost resembled to those of Group I, with normal SMSG and kidney features. Group III salivary glands showed lower MDA levels (17.47 ± 0.58) compared to the Amiodarone treated samples (24.17 ± 1.72) and greater CAT levels (60 ± 5.06) compared to the Amiodarone group (35.33 ± 2.73). Administration of CM significantly increased CAT (23.66 ± 1.36) and decreased MDA levels in kidney samples (5.53 ± 0.31) as compared to the Amiodarone group.

**Conclusions:**

BMSCs-CM has anti-inflammatory and anti-oxidative properties. Administering BMSCs-CM serve as cell-free therapy to treat the SMSGs and kidney damage caused by Amiodarone toxicity.

**Graphical Abstract:**

A diagram showing a graphical abstract for the present study.

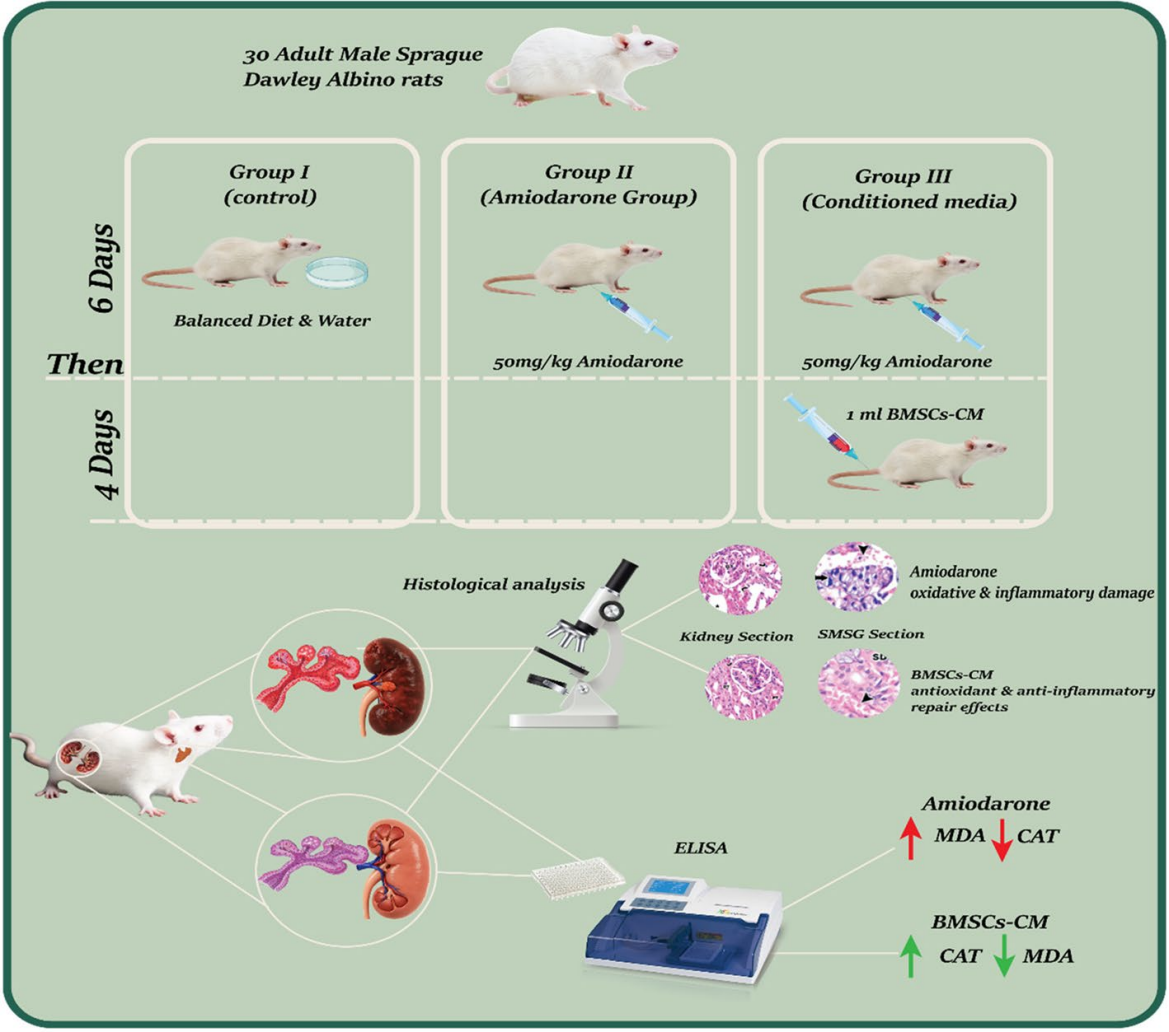

## Background

Amiodarone is a medication that used to treat the irregular heartbeats, atrial fibrillation and ventricular tachycardia. Because of its ease of administration, efficacy, low frequency of acute side effects, and mild inotropic side effects, it was originally thought to be an almost perfect antiarrhythmic drug [1]. Prolonged use may cause a wide range of adverse effects that impact several organs, some of which may be fatal [2]. Treatment with Amiodarone has been linked to toxicity and adverse effects in a number of organs, including the haematopoietic system, liver, heart, eye, skin, lung, nerve [3] and salivary glands [4]. Amiodarone's toxicity is typically dose-dependent, and cumulative effects can make it considerably more lethal [5]. Because of its high affinity for lipids, Amiodarone is tending to accumulate in various tissues, including the liver, lungs, skin, eyes, adipose tissue, muscles (especially the heart muscle), and the thyroid gland, this accumulation is responsible for its toxicity in different organs [6]. These adverse effects are frequently linked to oxidative stress, inflammation, and cellular damage [7], apoptosis, membrane destabilization and cytotoxic effects, both direct and indirect [5].

Treatment using mesenchymal stem cells (MSCs) is a novel approach to therapy with promising results. MSCs have shown encouraging results in treating a number of disorders, such as neurological, autoimmune, liver, heart, and kidney problems [8, 9]. Beyond their role in differentiation and tissue repair [10], the scientists hypothesized that these cells' protective function might be due to their potential antioxidant, anti-inflammatory, and anti-apoptotic properties [11], as well as their immunomodulatory effect [12]. A variety of disorders are presently being researched and evaluated using bone marrow derived MSCs (BMSCs) [13]. In certain organs, such as the heart, cartilage, brain tissue, and salivary glands, BMSCs demonstrated a protective function against injury [11, 14]. Culturing MSCs in vitro allow the release of a variety of bioactive molecules as cytokines, growth factors, and exosomes, into the surrounding medium. This mix is called conditioned medium (CM). The Mesenchymal stem cell conditioned media (MSC-CM) exerts its therapeutic effects based on the paracrine signaling mechanism of the released molecules that can influence nearby cells and tissues, promoting their ability to regenerate [15], downregulate pro-inflammatory cytokines, iNOS, fibrogenic growth factors [16], promote tissue healing and regeneration by modulating the immune response, minimizing inflammation and fibrotic effects, fostering the development of new blood vessels and nerve cells, and preventing cell death [17]. At the same time, they raised anti-inflammatory and anti-apoptotic molecules [18]. MSC-CM are being extensively studied for a number of regenerative therapeutic applications, including bone regeneration, myocardial infarction, stroke, wound healing, and hair growth [15].

Oral cavity is the only bodily part subjected to a wide range of environmental influences, including food, medications, air pollution, harmful lifestyle factors (such as alcohol consumption and tobacco smoke), and dental materials. The oral cavity's oxidative-reductive balance may be impacted by systemic oxidative stress [19]. Damage to secretory cells might result from the accumulation and aggregation of products of protein/lipid oxidation in the salivary glands [20, 21]. Research indicates that oxidative stress in children with kidney disease affects the oral cavity in addition to the kidneys, which results in increased oxidative damage to salivary proteins and alterations of the enzymatic and non-enzymatic antioxidant barrier [22, 23]. Saliva synthesis declines and protein release into saliva is disrupted by chronic renal failure. This could be because the salivary glands' parenchyma is damaged by the buildup of protein oxidation products, which causes hyposalivation [20, 24, 25]. Therefore, our study aimed to evaluate the toxic effects of Amiodarone on both SMSG and kidney and the possible therapeutic potential of BMSCs-CM.

## Methods

### Animal subjects and sample size

G*Power 3.1.9.2 software (Heinrich-Heine-Universität Dusseldorf, Germany) was used to calculate the sample size. To determine the necessary sample size, an a priori study using ANOVA (fixed-effects, special, main effects, and interactions) was conducted. Effect size (f = 0.40), power (power = 0.95), numerator (df = 4), and α error probability (0.05) were the input parameters. Three groups and a single assessment period served as the predictor factors [26]. In this study, thirty adult male albino rats, weighing 180–230 gm, between the ages of three and four months were used. Rats were obtained from the Animal House, Faculty of Medicine, Mansoura University, Egypt. All experimental procedures were performed under approved protocol of Mansoura University Animal Care and Use Committee (MU-ACUC), Egypt code number MU-ACUC (DENT.R.24.11.14). Only adult male rats were used in this study in order to minimize the biological variability associated with the estrous cycle in females, which can influence hormonal levels and, consequently, the structure and function of salivary glands and kidneys. This approach aimed to ensure more consistent and interpretable baseline data under controlled experimental conditions. The rats lived in a light-controlled environment with a 12:12 light–dark cycle, where the temperature was kept at 22ºC and the relative humidity was kept between 65 and 70%. They were given water and a commercial soft diet free access (ad libitum) allowing unrestricted access throughout the experiment. Every biosecurity and biosafety process for laboratory research was carried out in accordance with Alderman et al.'s recommendations [27].

Three equal and random groups (*n* = 10) of rats were created. Group I (Normal control): For six days, rats were provided a healthy diet and clean water; no medications were administered. Group II (Amiodarone Group): A daily intraperitoneal injection of Amiodarone (Sunny Pharmaceutical, 100 Fadan Industrial Zone, Badr City, Egypt) (Sanofi-Winthrop) at a dose of 50 mg/kg body weight per day for six days was administered [28]. Group III (conditioned media (CM) group): The rats were given Amiodarone according to the same protocol as group II. and they received 1 mL [29] of BMSC-CM by intravenous injection (IV) for four consecutive days after Amiodarone injection [30]. Based on earlier studies showing that short-term CM administration can significantly protect tissue and encourage functional recovery in acute organ injury models, the four-day CM treatment duration was selected [29, 30]. At the end of the experiment, animals were anesthetized using an intraperitoneal injection of ketamine (80 mg/kg) and xylazine (10 mg/kg) to ensure deep anesthesia prior to sample collection and surgical procedures. Euthanasia was then performed by cervical dislocation while the animals were fully unconscious. 150 ml blood sample was drawn from the caudal vein. Following centrifugation of the obtained blood samples, the serum was separated and tested for blood urea nitrogen and serum creatinine. Four-micron sections were prepared from kidney and submandibular salivary gland samples and stained with hematoxylin and eosin and Masson’s trichrome and immunohistochemical staining for caspase 3 staining. The submandibular salivary gland was selected for examination due to its predominantly serous acini and the presence of granular convoluted tubules, characteristics that render it more vulnerable to drug-induced toxicity, including that associated with amiodarone. The parotid gland which is exclusively serous was eliminated in order to preserve histological consistency and focus the scope of the current investigation. The levels of tissue malondialdehyde (MDA) and catalase (CAT) and serum interleukin 6 (IL-6) and TNF-α was performed by ELISA method.

### Bone marrow mesenchymal stem cells harvesting and isolation

As previously reported by Li et al., BMSCs were isolated using the direct adherence approach [31]. One flask was used to cultivate the isolated cells from a single rat. Three healthy male albino rats weighing between 100 to 120 g aging 3 to 4 weeks were put down via cervical dislocation. Following a 10-min immersion in 75% ethanol, the rats' femora and tibiae were separated under sterile conditions. The removed bones were submerged in low blood glucose Dulbecco's modified eagle's medium (L-DMEM, Hyclone, Logan, UT, USA) with 100 μg/mL streptomycin and 100 U/mL penicillin. The joint capsules at the ends of each bone were removed, and the diaphysis was separated. In order to routinely clean the bone marrow cavity with the antibiotic-supplemented L-DMEM solution, the cells were harvested in sterile petri dishes using an aseptic disposable syringe. The resultant cell suspension was centrifuged by (Hettich ZENTAIFUGEN D-78532 Tuttlingen, Germany) centrifuging device at 250 × g for 5 min at 26 °C, and then it was once again washed in L-DMEM supplemented with 10% fetal bovine serum. The cells were transferred to a 25 cm^2^ plastic culture flask and incubated in 5% CO2 at 37 °C after being resuspended in complete medium containing 90% L-DMEM, 10% fetal bovine serum, 100 μg/mL streptomycin, and 100 U/mL penicillin.

### Immunophenotype characterization of BMSCs

BMSCs from passage three were rinse with phosphate buffer saline (PBS). After that, 0.5 ml of PBS was used to resuspend the cells. Using fluorescein isothiocyanate as the fluorophore, rabbit polyclonal anti-CD90, mouse monoclonal anti-CD105, and rabbit polyclonal anti-CD45 antibodies (Abcam, Cambridge, United Kingdom) were added separately and incubated for 30 min at 4 °C in the dark. After being washed in PBS, labelled BMSCs were centrifuged by (Hettich ZENTAIFUGEN D-78532 Tuttlingen, Germany) centrifuging device for five minutes at 2,000 rpm and then resuspended in PBS. Labelled BMSCs were examined for immunophenotypes using a BD Accuri C6 flow-cytometer that has the BD Accuri C6 program software installed.

### Collection and preparation of BMSCs conditioned media (BMSCs-CM)

BMSCs-CM were collected and prepared using the same technique as described by [32]. It was produced by culturing newly trypsinised BMSCs of passage 2 for eight hours in an incubator with 5% CO2 in serum-deprived fresh DMEM (Gibco) that included 10% fetal bovine serum and 1% penicillin–streptomycin. To get rid of any detached cells, the cell supernatant was collected from the tissue culture vessels after the incubation period and centrifuged by (Hettich ZENTAIFUGEN D-78532 Tuttlingen, Germany) centrifuging device for five minutes at 1800 rpm.

### ELISA assay of BMSCs-CM (characterization of BMSCs-CM)

ELISA was carried out using the pooled BMSCs-CM in accordance with the manufacturer's instructions to determine the levels of interleukin 10 (IL-10) and platelet derived growth factor (PDGF). The quantitative enzyme immunoassay technique was used with specific antibodies for the target PDGF or IL-10 proteins. Antibodies were pre-coated onto a 100 µL microplate. Any target protein that was present was bound by the immobilized antibody and the wells were pipetted with samples and any unbound materials had been removed. A biotin-conjugated antibody was added to the wells that was specific for either PDGF (LS Bio, catalogue number Ls-F27550) or IL-10 (Rat Interleukin ELISA kit, MyBiosource, Cat. no. MBS269138). The wells were cleaned and then treated with avidin-conjugated horseradish peroxidase. A stop solution was added to halt the color development, and a microplate reader that could measure absorbance at 450 nm with the correction wavelength set at 570–630 nm was used to measure the color's intensity. A standard curve was compared to the plotted quantitative concentration findings of PDGF and IL-10. Following the addition of a substrate solution, the signal was detected and was proportionate to the target's concentration. For PDGF, the detection range was 100–2500 pg/mL, while for IL-10, it was 15.6 pg/mL. For rat PDGF-AB, the lowest detectable dose was usually less than 1 pg/mL, but for IL-10, it was usually as high as 5 pg/mL. The lowest protein concentration at which this assay could distinguish itself from zero was known as the sensitivity or lower detection.

### Plasma creatinine and urea concentrations

A colorimetric technique based on the Jaffe´ reaction (SPINREACT, S.A./S.A.U.Ctra.Santa Coloma, 7 E-17176 SANT ESTEVE DE BAS (GI) SPAIN) was used to detect the amounts of plasma creatinine and urea [33]. An enzymatic approach (Hitachi 917, Roche Diagnostics, Mannheim, Germany) was used to measure the levels of urea. The assay is based on the reaction of creatinine with sodium picrate. Creatinine reacts with alkaline picrate forming a red complex. The time interval chosen for measurements avoids interferences from other serum constituents.

### Evaluating the salivary function by measuring salivary amylase

The salivary flow rate was not measured because pilocarpine, required to stimulate salivation in experimental animals, was not available in Egypt at the time of the experiment. Hence the salivary flow rate was not recorded. Rather, salivary amylase levels were measured as a functional indication of glandular activity using an ELISA technique. Using Assay Genie Rat AMY1 ELISA Kit* (*Catalogue Code: RTES0115, Sensitivity: 0.94 ng/mL& Detection Range: 1.56—100 ng/mL)*.* The SMSG was dissected, cleaned with saline, minced into small pieces and rinsed in ice-cold PBS (0.01 M, pH = 7.4) to remove excess blood thoroughly. Tissue pieces were weighed and then homogenized in PBS (tissue weight (g): PBS (mL) volume = 1:9) with a glass homogenizer on ice. The homogenates are then centrifuged for 5 min at 5000 × g to get the supernatant [34].

### Determination the levels of malondialdehyde (MDA) and catalase (CAT) in SMSG and kidney tissues

Malondialdehyde (nmol/mg) and catalase (mg/dL) as indicators of lipid peroxidation were measured using an ELISA. Following the manufacturer's recommendations, the right kidney and SMSG halves were cleaned using ice-cold phosphate-buffered saline to remove any remaining blood. To stop oxidation and proteolysis, butylated hydroxytoluene and a proteolysis inhibitor were added to the samples. Following homogenization, the samples were kept overnight at −20 °C in 20 mL of 1 × PBS. Following two freeze–thaw cycles, the homogenate was centrifuged for five minutes at 5,000 × g to break the cell membranes and eliminate any particle elements. Following separation, the supernatant was kept at −20 degrees Celsius. A Ray to Stat Fax 3200 microplate reader was used, and rat MDA (Rat Malondialdehyde (MDA) ELISA Kit, Catalogue Code: RTEB1739) and catalase (Rat Catalase (Cat), Catalogue Code: RTEB0173) ELISA test kits were supplied by Assay Genie. Before being used, the obtained samples were allowed to reach room temperature after being held at −80 °C. Tissue samples, ranging in volume from 50 to 100 μL per well, were put to the microplate's experimental wells as directed by the manufacturer of each kit. The antibody mixture was then added to each of the experimental and control wells in amounts ranging from 50 to 100 μL. After applying an adhesive seal, the microplates were left to stand at room temperature for two hours before being agitated on a microplate shaker. The experimental wells were gently washed three times with 350 μL of wash buffer per well. After adding 200 μL/well of the antibody conjugate, the plate was shaken for an hour at room temperature in the dark. The shaker plate was incubated for 30 min after 200 μL of the substrate solution was added to each well. After adding fifty microliters of stop solution, each well-turned yellow. The absorbance was measured at 450 nm within 30 min of applying the stop solution [35].

### Measuring the levels of serum IL-6 & TNF-α as an inflammatory marker

Following collection, blood samples were centrifuged, and the resulting serum was kept at 80 degrees Celsius. At the end of the study, serological analyses were performed on all serum samples. ELISA assays were performed to measure serum levels of IL-6 and TNF- α using two monoclonal antibodies that were specific to different antigenic determinants on rat IL-6 (Quantikine® Colorimetric Sandwich ELISA Kits, Catalog Number R6000B) and rat TNF-α (CUSABIO®, Catalog Number. CSB-E11987r) [36].

### Histological examination

The kidneys and SMSG specimens were immersed in a fresh 4% formalin solution and left in paraffin for 48 h. After that, it was dehydrated, cleaned, and penetrated with melted paraffin wax. It was then embedded in hard paraffin and sectioned. Sections were dewaxed with xylene with thickness from 3 to 5 μm. Sections measuring three to five microns were produced for histological staining with H&E [14, 35] and Masson’s trichrome stain [37] and they were examined under an Olympus microscope. From every sector, three areas were chosen at random for inspection. Histological evaluation was done qualitatively for H&E-stained sections to evaluate tissue architecture integrity, inflammatory infiltration, and cellular degeneration. Collagen deposition was evaluated semi-quantitatively using ImageJ software by computing the percentage area of collagen in five randomly chosen fields per section for Masson's trichrome-stained sections, where collagen fibers appeared as blue patches. An experienced histopathologist who was blind to the experimental grouping performed all histological exams.

### Caspase 3 immunostaining

Caspase 3 antibodies, an indicator of programmed cell death, were used in immunohistochemical staining. In rehydrated paraffin-embedded section, endogenous peroxidase activity was inhibited by immersing them in a 3% hydrogen peroxide (H2 O2) solution in methanol at 23 °C for 15 min. Subsequently, the sections were microwaved in a citrate buffer (pH 6.0) for 9 min. Following this, a polyclonal rabbit anti-active caspase 3/CPP32 antibody (Diagnostic BioSystems, RP96) in PBS was applied for 60 min at 23°C. Next, a secondary biotinylated goat anti-rabbit antibody was used for 30 min. The slides were then incubated, developed with diaminobenzidine, and counterstained with hematoxylin [38].

### Computer‑assisted digital image analysis

An Olympus® digital camera with a 40 × objective and a 1/2 × photo converter was used to photograph the slides. Using Video Test Morphology® software (Russia), this features a unique built-in system for counting items and measuring area and percentage, the images were examined on a computer with an Intel® Core I5® processor.

### Statistical analysis

SPSS software, version 26 (SPSS Inc., PASW statistics for Windows version 26. Chicago, Illinois: SPSS Inc.), was used to analyze the data. Following a Shapiro–Wilk test to verify normality, quantitative data were presented using mean ± standard deviation for normally distributed data. Significance of the obtained results was judged at the (≤ 0.05) level. One Way ANOVA test was used to compare more than 2 independent groups with Post Hoc Tukey test to detect pair-wise comparison.

## Results

### Characterization of BMSCs

**Fig. 1:**
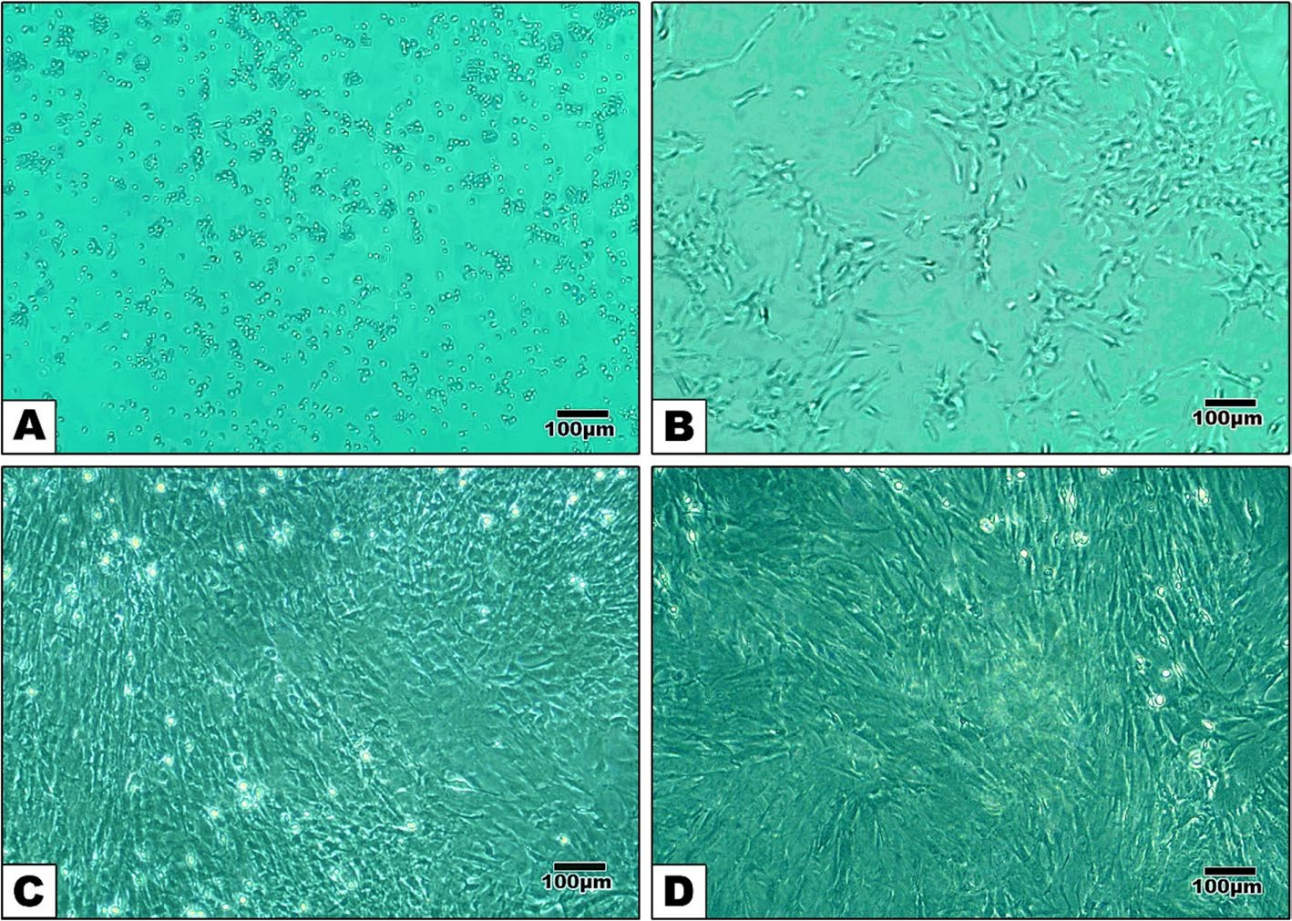
Inverted microscopy images of BMSCs reveal (**A**) numerous spherical cells on the second day of isolation, (**B**) number of fibroblast-like cells attached to the flask on the 4th-day post-isolation, (**C**) proliferated by day 7 (**D**) Cell confluence was observed on the 16th day (Scale bar = 100µm)

Adherence separation was the technique used to isolate BMSCs. In along with confluence rate, stem cell adhesion, division, and growth were assessed using an inverted phase contrast microscope. In cell culture biology, confluence is the percentage of a culture dish's surface that is covered by adherent cells (Fig. 1).

On second day of isolation: Numerous spherical cells appeared.

On the fourth day of isolation: The rounded cells started to develop spindle fibroblast-like cells. These cells stuck to the flask during the first culture with the first change of the media, whereas the non-adherent cells were thrown away with the old media.

On the seventh day of isolation: The formation of colonies with fibroblast-like cells in between was observed.

On the sixteenth day of isolation: The monolayer cell was formed and the number of cells was increased. After achieving 90% confluence, the adherent cells were prepared for passage.

### Immunophenotype characterization

**Fig. 2:**
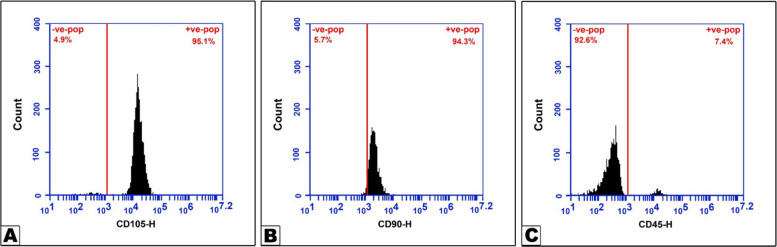
Flow cytometry chart with single-parameter histograms for CD105 (**A**), CD90 (**B**), and CD45 (**C**)

From cell culture at passage 3, the immunophenotype's characterization was evaluated. The surface markers of BMSCs in the cultivated specimen exhibited a positive reaction for the mesenchymal markers CD105 (95.1%) and CD90 (94.3%), and a negative or weakly positive reaction for the standard hematological marker CD45 (7.4%). Together, these results confirmed that there were no hematopoietic cells in the culture and that the cells were indeed ectomesenchymal in origin (Fig. 2).

### ELISA assay results of BMSCs-CM

In the pooled BMSCs-CM, PDGF and IL-10 were identified. Both PDGF and IL-10 were found in high concentrations, 264.5 and 287.76 pg/mL and 247.2 and 267.72 pg/mL, respectively.

### Serum creatinine and blood urea nitrogen

**Fig. 3:**
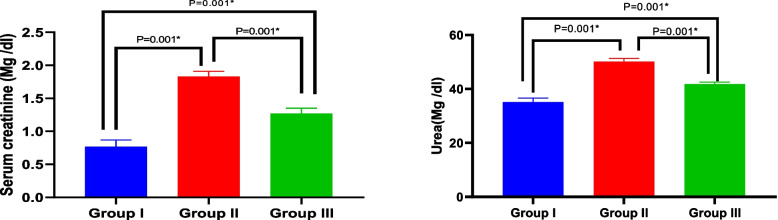
Bar graph shows levels of serum creatinine and urea between studied groups

Creatinine and blood urea nitrogen levels significantly increased (1.83 ± 0.08 & 50.17 ± 1.17) in the biochemical serum test performed on Amiodarone treated rats. When BMSCs-CM were injected, the creatinine and blood urea levels dropped (1.27 ± 0.08 &41.83 ± 0.75) in comparison to the Amiodarone group. An ANOVA test showed that there was overall significant difference between all groups regarding creatinine and blood urea nitrogen levels with *p* value ≤ 0.001 (Table 1, Fig. 3).

**Table 1:** Comparison of serum creatinine and urea between studied groups

	Group I (*n* = 6)	Group II (*n* = 6)	Group III (*n* = 6)	Test of significance	Post Hoc Tukey		
					P1	P2	P3
Serum creatinine (mg/dL)	0.767 ± 0.10	1.83 ± 0.08	1.27 ± 0.08	F = 213.61 *P* < 0.001*	0.001*	0.001*	0.001*
Urea (mg/dL)	35.17 ± 1.47	50.17 ± 1.17	41.83 ± 0.75	F = 247.96 *P* < 0.001*	0.001*	0.001*	0.001*

One Way ANOVA test, *statistically significant, p1: difference between group I versus II, p2: difference between group I versus III, p3: difference between group II versus III

### Salivary amylase

**Fig. 4:**
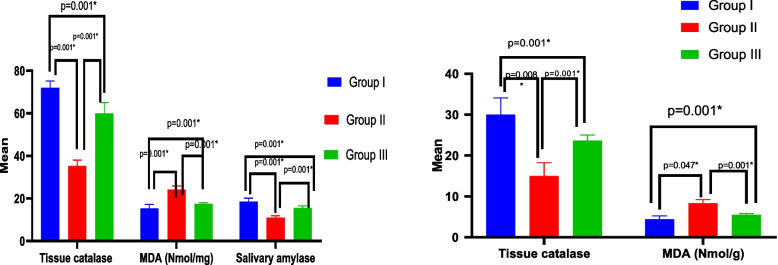
Bar graph showing levels of salivary and kidney markers between studied groups

Amylase activity in SMSGs was reduced by Amiodarone treatment (11 ± 0.89). However, there was a notable rise in amylase activity (15.50 ± 1.05) in the CM group. An ANOVA test showed that there was overall significant difference between all groups regarding salivary amylase with *p* value = 0.001 (Table 2, Fig. 4).

**Table 2:** Comparison of salivary and kidney markers between studied groups

	**Group I**	**Group II**	**Group III**	**Test of significance**	**P1**	**P2**	**P3**
Salivary Tissue catalase (mg/dL)	72 ± 3.22	35.33 ± 2.73	60 ± 5.06	F = 144.72 P = 0.001*	0.001*	0.001*	0.001*
Salivary MDA (nmol/mg)	15.33 ± 1.86	24.17 ± 1.72	17.47 ± 0.58	F = 185.53 *P* = 0.001*	0.001*	0.001*	0.001*
Salivary amylase (ng/mg)	18.50 ± 1.64	11 ± 0.89	15.50 ± 1.05	F = 55.76 *P* = 0.001*	0.001*	0.002*	0.001*
Kidney catalase (nmol/mg)	30 ± 4.09	15 ± 3.22	23.66 ± 1.36	F = 35.12 *P* = 0.001*	0.001*	0.008*	0.001*
Kidney MDA (nmol/g)	4.40 ± 0.86	8.30 ± 0.91	5.53 ± 0.31	F = 43.46 *P* = 0.001*	0.001*	0.047*	0.001*

### Levels of MDA and catalase in SMSG and kidney tissues

Amiodarone treated samples of SMSG and kidney showed significantly elevated MDA levels and reduced CAT compared to the control group. The samples that were treated with BMSCs-CM showed lower levels of MDA and higher levels of CAT than the Amiodarone group. The MDA and CAT levels in both the kidney and SMSG groups showed that there was overall significant difference between all groups according to an ANOVA test with a p value of 0.001. When compared to the control group, the renal catalase protein abundance was significantly lower in the Amiodarone group, indicating kidney damage. The Amiodarone induced catalase inhibitory activity was stopped by the injection of BMSCs-CM (Table 2, Fig. 4).

### Serum IL-6 and TNF-α

**Fig. 5:**
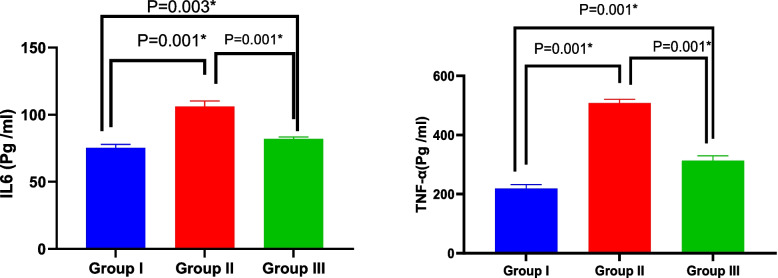
Bar graph show serum levels of IL 6 & TNF-α markers between studied groups

Experimental rats' serum IL-6 and TNF-α levels were assessed utilizing ELISA analysis. The conditioned media group (82 ± 1.41) and the control group (75.33 ± 2.58) had similar average serum levels of IL-6, but the Amiodarone group had greater levels (106.17 ± 3.97). The Amiodarone group had higher serum levels of TNF-α (508.16 ± 12.36) pg/mL than the CM group (313.17 ± 16.36). With a *p* value ≤ 0.001, an ANOVA test revealed that there was an overall significant difference in IL-6 and TNF-α between all groups (Table 3, Fig. 5).

**Table 3:** Comparison of serum IL 6 & TNF-α markers between studied groups

	Group I (*n* = 6)	Group II (*n* = 6)	Group III (*n* = 6)	Test of significance	Post Hoc Tukey		
					P1	P2	P3
IL6 (pg/mL)	75.33 ± 2.58	106.17 ± 3.97	82 ± 1.41	F = 193.89 *P* < 0.001*	0.001*	0.003*	0.001*
TNF-α(pg/mL)	219.33 ± 12.72	508.16 ± 12.36	313.17 ± 16.36	F = 670.72 *P* < 0.001*	0.001*	0.001*	0.001*

### Hematoxylin and eosin stain results of salivary gland

**Fig. 6:**
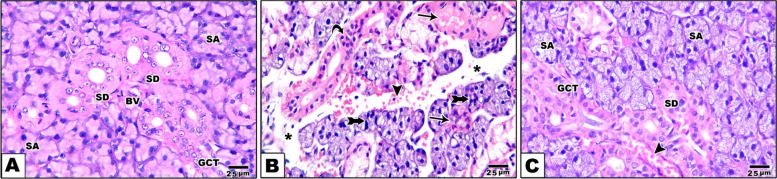
The photomicrograph of salivary gland sections stained with H&E illustrates the typical histological structure of a rat's submandibular salivary gland. It features densely packed serous acini (SA) lined with eosinophilic pyramidal cells and basophilic round nuclei positioned basally, along with normal striated ducts (SD), blood vessels (BV), and granular convoluted tubules (GCT) in the control group [**A**]. In the Amiodarone group, the section reveals significant duct degeneration (arrow), a loss of classical architecture, and an increase in interstitial connective tissue spaces between severely degenerated serous acini (star). The lining cells exhibit nuclear abnormalities and varied shapes (tailed arrow), with disrupted continuity of the acinar cell membranes, pyknotic nuclei, and cytoplasmic vacuoles (curved arrow), as well as interacinar blood capillary hemorrhage (arrowhead) [**B**]. The salivary glands of the CM treated group appear relatively preserved, with restoration of their histological and architectural integrity, with closely packed serous acini (SA), striated ducts (SD), and granular convoluted tubules (GCT), showing only minor nuclear pyknosis and macro vacuoles in the acinar and ductal cells. The interacinar blood capillary appears normal (arrowhead) [**C**]. Scale bar 25 µm “H&E × 400”

When the SMSGs of control rats were examined under a light microscope, the duct system and acini showed normal histological architecture. Normal acini were primarily serous and bordered with pyramidal cells and spherical open faced basal nuclei. The SMSGs of group II displayed cytoplasmic vacuolation at acinar and ductal cells, striated duct lining epithelial degradation, and luminal shedding. Additionally, interacinar blood capillary hemorrhage was observed. Significant acini and duct degeneration, leaving empty spaces in some places. Lining cells with nuclear abnormalities, thickening and enlargement of interstitial spaces, and loss of the gland's typical architecture with a changed appearance. In comparison to the amiodarone group, the CM group displayed more acini and ducts with normal nuclei, fewer vacuoles, normal interacinar blood capillary and clearer lobular structures. According to histological investigation, these findings were apparent signs of improvement and healing (Fig. 6).

### Hematoxylin and eosin stain results of kidney

**Fig. 7:**
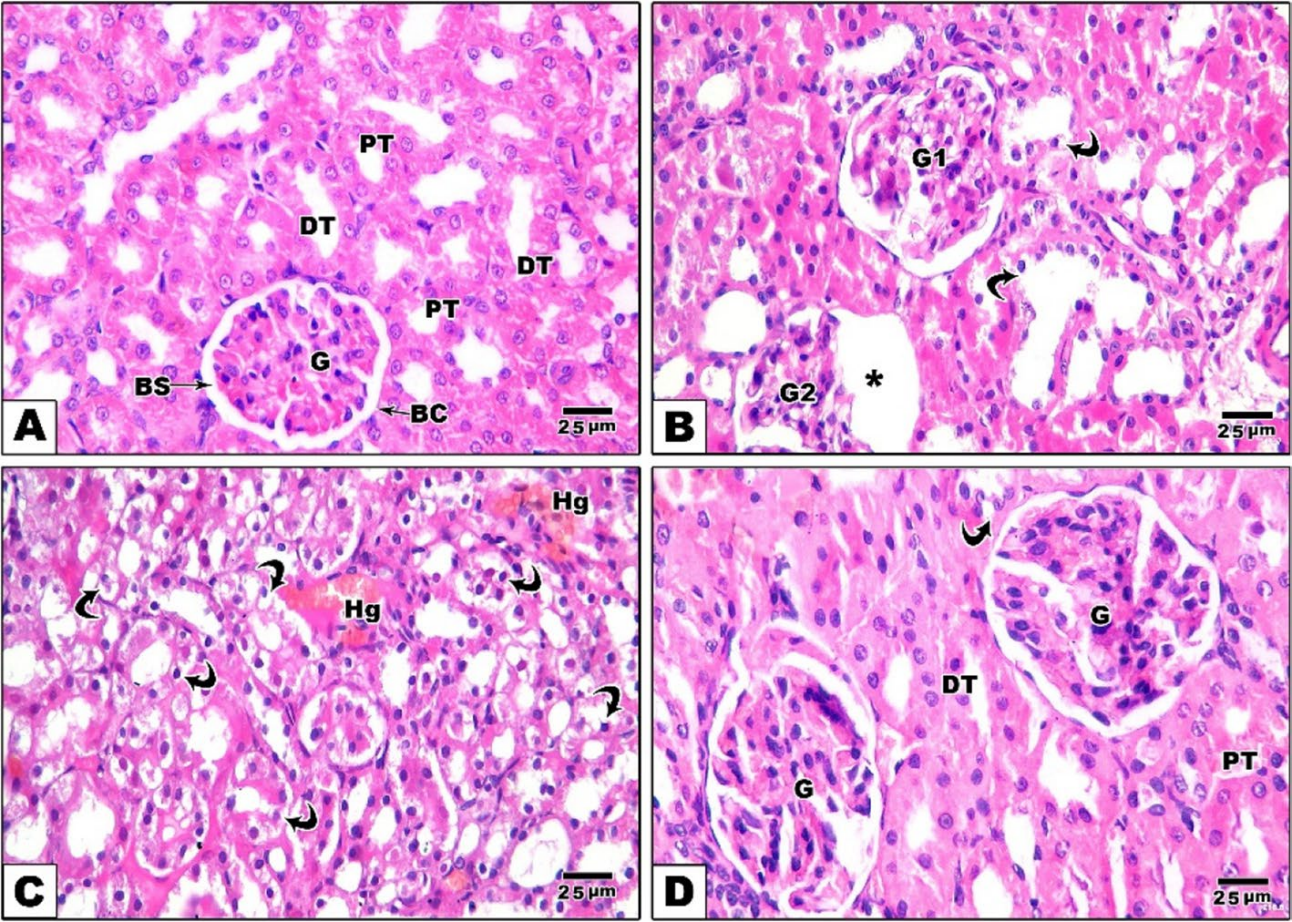
Histopathological analysis of renal cortex sections under various experimental conditions (H&E staining). **A** A section from the control group displays the normal histological structure of renal cortex. The glomerulus (G) is surrounded by a distinct Bowman’s space (BS) and Bowman’s capsule (BC). Both proximal tubules (PT) and distal tubules (DT) are intact with their epithelial lining preserved. **B** A section from the Amiodarone group reveals glomerular and tubular damage; G1 = disrupted glomerular capillaries, G2 = shrinkage of glomerular capillaries with dilated Bowman’s space (asterisk) and curved arrow = vacuolar degeneration of renal tubules. **C** Another section from the Amiodarone group shows excessive vacuolar degeneration with dark stained nuclei of epithelial lining of renal tubules (curved arrow) and hemorrhagic areas (Hg). **D** A section from the CM group shows relatively preserved glomeruli (G) with mild tubular changes (curved arrow) and improved renal architecture. Scale bar 25 µm. “H&E × 400”

When it comes to assess kidney sections, control rats showing normal architecture of renal cortex with normal glomerular capillaries surrounded by Bowman’s capsule and narrow Bowman’s space and normal epithelial lining of both proximal and distal convoluted tubules. In rats of Amiodarone group, the kidney shows disruption and shrinkage of glomerular capillaries with dilated Bowman’s space and multiple hemorrhagic areas. Also, excessive vacuolar degeneration with darkly stained nuclei of epithelial lining of renal tubules is evident resulting in tubular dilation. In rats of CM group, the kidney shows improved renal architecture with relatively normal glomeruli and cortical tubules except few tubular changes (Fig. 7).

### Histological analysis of Masson’s trichrome

**Fig. 8:**
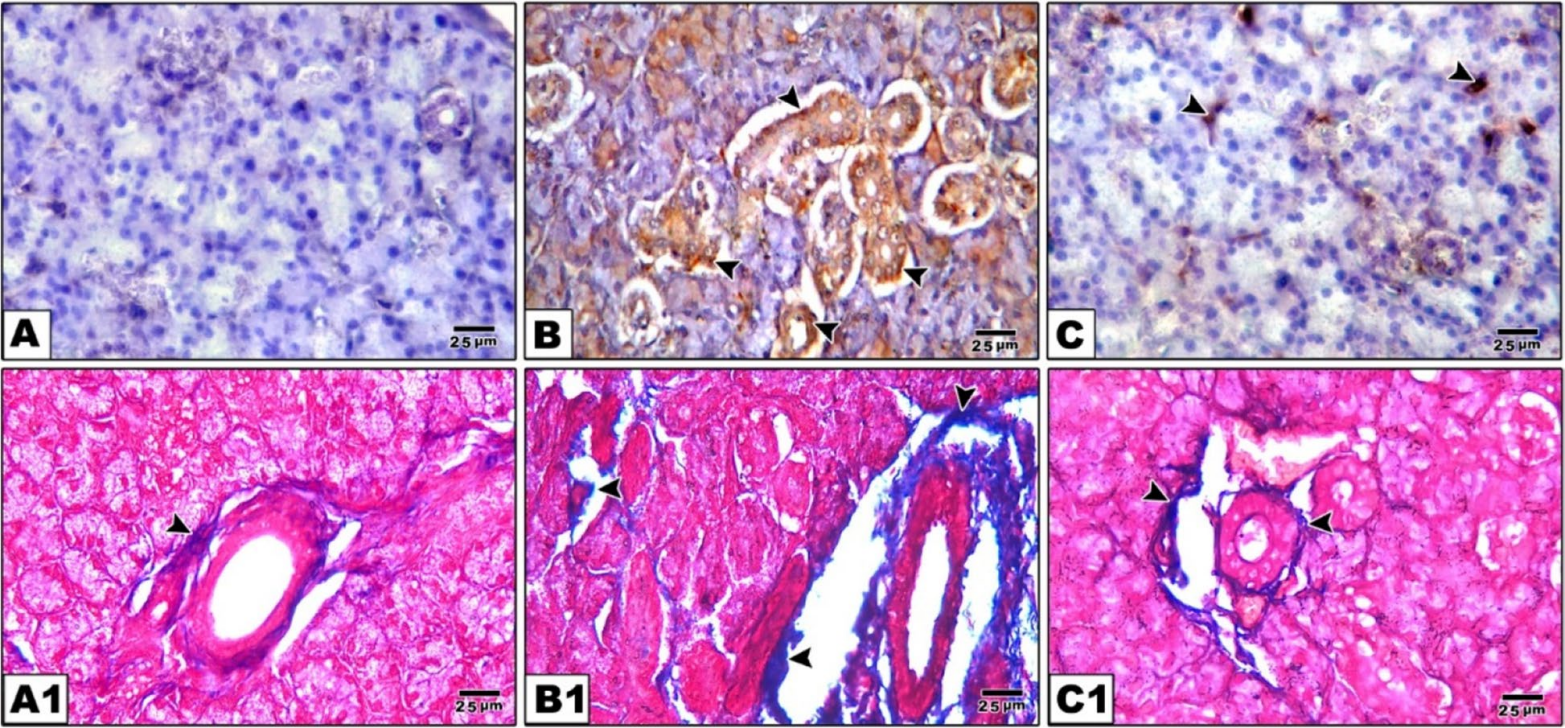
Caspase 3 immunoexpression and collagen deposition (Masson’s trichrome staining) in submandibular salivary glands from various experimental groups. **A**–**C** Caspase-3 immunohistochemistry (brown cytoplasmic staining). **A** The control group displays weak caspase 3 immunoreactivity in both acinar and ductal cells. **B** The group treated with Amiodarone shows strong positive caspase 3 expression (arrowheads) in the cytoplasm of acinar and ductal cells. **C** The CM treated group demonstrates a moderate decrease in caspase 3 immunoexpression (arrowheads) compared to the Amiodarone group. A1–C1 Masson’s trichrome staining reveals collagen deposition (blue staining). A1 The control group has minimal collagen fibers deposition (arrowhead) surrounding ducts and blood vessels. B1 The Amiodarone group exhibits excessive collagen fiber accumulation (arrowheads), particularly around ducts, blood vessels, and between acini. C1 The CM treated group shows moderate collagen deposition (arrowheads), less than the Amiodarone group, suggesting reduced fibrotic changes. Scale bar 25 µm. “Immuno × 400”

**Fig. 9:**
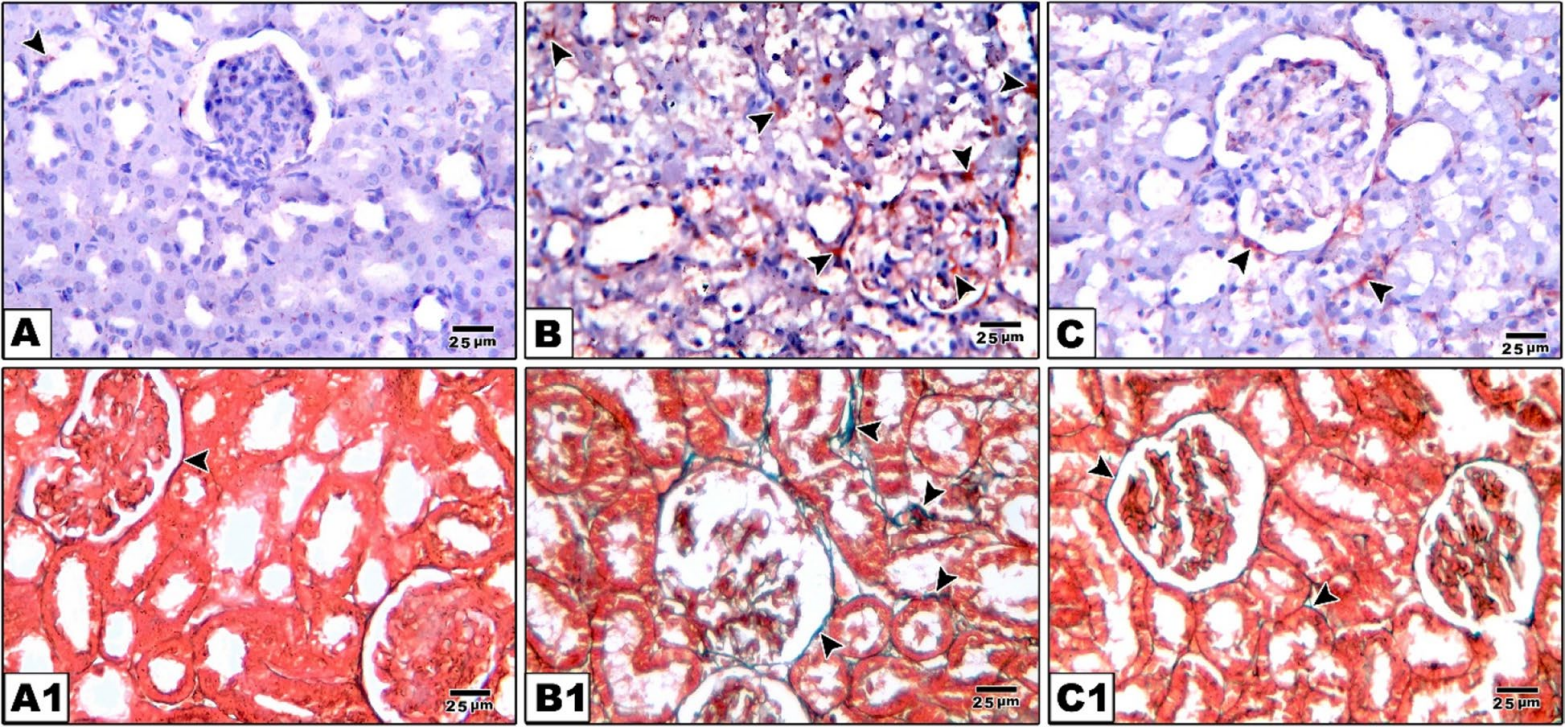
Caspase-3 immunoexpression and Masson’s trichrome staining of kidney tissues in the experimental groups. **A**–**C** Caspase-3 immunohistochemical staining: **A** Control group showing negative caspase-3 immunoreactivity, indicating absence of apoptotic activity. **B** Amiodarone-treated group displaying strong positive cytoplasmic caspase-3 immunoexpression (arrowheads) in renal tubular epithelium and glomeruli, indicating marked apoptosis. **C** CM-treated group showing moderate caspase-3 immunopositivity (arrowheads) compared to the Amiodarone group. A1–C1 Masson’s trichrome staining (blue staining for collagen fibers): A1 Control group showing minimal collagen deposition (arrowhead) around blood vessels and interstitium, consistent with normal extracellular matrix. B1 Amiodarone-treated group showing marked increase in collagen fiber deposition (arrowheads) around renal tubules and vasculature. C1 CM-treated group showing reduced collagen deposition (arrowheads) compared to the Amiodarone group. Scale bar 25 µm “ × 400”

**Fig. 10:**
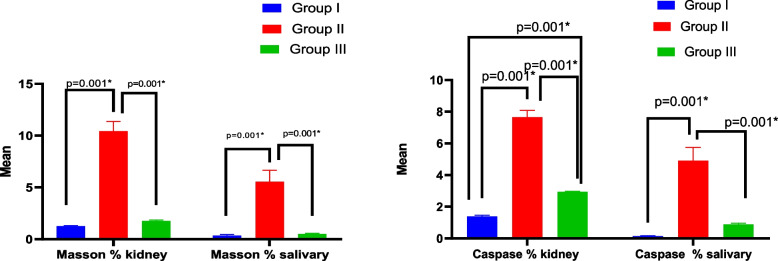
Bar graphs show caspase 3 & Masson trichrome expression in kidney and salivary gland

We used Masson's Trichrome staining to look more closely at collagen type I deposition in the tissues. Blue or green color staining were evident representing collagen fibers. Collagen fibers in the lamina propria of SMSG were excessive, looser and less organized in the Amiodarone group (5.56 ± 1.10) especially around ducts, blood vessels and between acini reflecting fibrosis, which is significant sign of degeneration. Control group (0.35 ± 0.1) show minimal collagen fibers around ducts and blood vessels, indicating normal extracellular matrix composition, while CM treated group (0.493 ± 0.06) exhibiting moderate collagen deposition, less than the Amiodarone group, indicating reduced fibrotic changes suggesting protective effects of CM treatment. Control kidney tissue (1.26 ± 0.05) showing minimal collagen deposition around glomeruli and tubules indicating normal extracellular matrix. Amiodarone treated group (10.42 ± 0.95) showing marked increase in collagen fibers around renal tubules and glomeruli, reflecting fibrosis. CM treated group (1.75 ± 0.09) showing reduced collagen fiber deposition compared to the Amiodarone group, indicating attenuation of fibrotic changes. An ANOVA test showed an overall significant difference between all groups with *p* value = 0.001 (Figs. 8, 9, 10 and Table 4).

**Table 4:** Comparison of caspase 3 & Masson trichrome expression in kidney and salivary between studied groups

	Group I (***n*** = 6)	**Group II** **(*n* = 6)**	**Group III** **(*n* = 6)**	**Test of significance**	**P1**	**P2**	**P3**
Caspase % kidney	1.39 ± 0.08	7.65 ± 0.44	2.94 ± 0.039	F = 625.70 P = 0.001*	0.001*	0.001*	0.001*
Caspase % salivary	0.145 ± 0.026	4.90 ± 0.85	0.884 ± 0.08	F = 108.41 P = 0.001*	0.001*	0.139	0.001*
Masson % kidney	1.26 ± 0.05	10.42 ± 0.95	1.75 ± 0.09	F = 346.12 P = 0.001*	0.001*	0.453	0.001*
Masson % salivary	0.35 ± 0.1	5.56 ± 1.10	0.493 ± 0.06	F = 86.02 P = 0.001*	0.001*	0.948	0.001*

### Immunohistochemical analysis of caspase 3

Apoptosis was assessed by evaluating caspase 3 immunostaining in the tissues of the three groups. The staining reaction was seen in the cytoplasm of the entire salivary glandular component. In the control and conditioned media groups, its expression displayed a mild cytoplasmic reaction in the ductal and acinar epithelium (0.145 ± 0.026& 0.884 ± 0.08) respectively. The cytoplasmic and nuclear immunoreactions of ductal cells were moderately to strongly positive in the Amiodarone group (4.90 ± 0.85). In terms of the kidney's immunohistochemical reactivity to caspase 3 antibody, the control (1.39 ± 0.08) and CM (2.94 ± 0.039) groups displayed mild immunoreactivity against caspase 3 antibody, which were mostly present in the renal corpuscles and the lining epithelium of the renal tubules. Meanwhile, the Amiodarone group (7.65 ± 0.44) demonstrated strong positive cytoplasmic caspase 3 immunoexpression in renal tubular epithelium and glomeruli, indicating extensive apoptosis which was mostly expressed within the renal tubule lining epithelium. An ANOVA test showed that there was overall significant difference between all groups regarding caspase 3 immunostaining with *p* value = 0.001 (Figs. 5, 6 and Table 4).

## Discussion

Amiodarone is a class III antiarrhythmic drug that frequently used to treat ventricular and atrial arrhythmias [39]. Although it is a powerful antiarrhythmic drug, the wide use of this medication has led to the occurrence of adverse effects, as pulmonary toxicity, hepatotoxicity, thyroid dysfunction, and others [40]. Because engrafted MSCs do not survive over the long term, recent research indicates that the benefits of MSC therapy may be due to their secreted factors [41]. There may be significant potential benefits to using MSCs-CM over living cells in terms of production, handling, storage, product shelf life, and their potential as ready to use bio therapeutics [42]. It has been shown that MSCs-CM are adequate to considerably ameliorate a number of pathophysiological biomarkers and to be successful in transplanting the corresponding MSCs in a variety of animal models. Numerous diseases including lung injury, cerebrovascular disease, spinal cord injury and others have been treated with MSCs-CM [43]. Remarkably, MSCs-CM have been shown to enhance renal function and structure while diminishing Amiodarone induced renal damage [44]. However, the results obtained using the CM are still debated. Therefore, the purpose of this study was to investigate the harmful effects of Amiodarone on the SMSGs and kidneys and as well as the potent antioxidant and anti-inflammatory effects of BMSCs-CM.

The BMSCs lack the hematopoietic stem cell markers CD34, CD14, and CD45 and express the MSC markers CD90, CD44, CD29, CD106, CD73, and CD105 [45]. In our study, three cell surface markers were most prominent: the presence of CD105 and CD90, and the absence of CD45. Using ELISA analysis, we also discovered that the BMSCs-CM samples had high levels of PDGF and IL-10. Previous studies of the factors present in MSC-CM using cytokine array analysis indicated that numerous cytokines and chemokines are present in MSC-CM and that these factors can influence different types of cell behavior [46, 47].

Creatinine and blood urea nitrogen are the two most significant pathological indicators of acute renal damage. Measuring serum concentration of waste products is their most common detection method [48]. Hence, biochemical analysis of these materials was done on the serum collected from the animals under study. The results of our study revealed that a considerable increase in the levels of creatinine and blood urea nitrogen in the animals treated with Amiodarone. When BMSCs-CM were injected, the creatinine level dramatically dropped in comparison to the Amiodarone group. Our results are in agreement with Morales AI et al. [28] who stated that to assess a potential nephrotoxic effect, a dose of 50 mg/kg/day could be used in the experimental rat model. This dosage of Amiodarone caused a significant rise in serum creatinine, which is consistent with tubular changes, proving that Amiodarone damages rats' kidneys. Also, Sakr SA et al. [49] found that Amiodarone caused increase level of both serum creatinine and urea. Serum creatinine increased in 30 patients who got Amiodarone over the course of a year. According to another prospective trial done by Pollak PT et al. [50], there was a strong association between the Amiodarone dosage and serum creatinine. Abouelkheir M et al. [51] also demonstrated that the creatinine level dropped considerably in the CM group when compared to the cisplatin group. This suggests that the MSCs-CM may be able to stop renal damage from progressing due to their anti-inflammatory and anti-apoptotic properties. Ogata K et al. [52] also confirmed and investigated that CM of the dental pulp stem cells had an anti-apoptotic effect on the SMSGs. They assessed the kidneys of MRL/lpr mice, and the findings indicated that CM improved the kidneys (e.g. creatinine level), reduced the expression of IL-6 and increased the expression of IL-10.

Salivary biomarkers have become common in research as they have proven superiority in testing biomarkers in blood [53]. α-amylase is the most abundant digesting enzyme protein in human saliva [54]. According to our findings, Amiodarone reduced amylase activity, but the BMSCs-CM treated group's salivary amylase levels significantly increased. These findings are consistent with those of Ikeno T et al. [34] who reported that amylase activity in serum and saliva was elevated by both cholinergic and adrenergic drugs. Acetylcholine enhanced the parotid gland's release of amylase into the bloodstream as well as its secretion into the ducts. Amiodarone suppresses Acetylcholine by suppressing the activity of the K Acetylcholine channel and/or related GTP-binding proteins, which in turn decreases amylase activity [55]. On the other hand**,** Horie H et al. [56] illustrates the beneficial impacts of adipose-derived stem cell and their derivatives on raising amylase activity.

Malondialdehyde is the final product of peroxidation of the polyunsaturated fatty acids in cells. Its level is common marker for oxidative stress. An increase in free radicals causes overproduction of MDA [57]. Antioxidant enzymes as catalase and superoxide dismutase (SOD) are the first line of defense against oxidative stress [58]. In the present study, the Amiodarone treated group had much greater MDA levels and lower CAT levels than the BMSCs-CM group which had significantly diminished MDA levels and restored CAT levels in both salivary gland and kidney samples suggesting their anti-oxidative properties preventing tissue damage induced by oxidative stress. Amiodarone causes oxidative stress and generated higher levels of reactive oxygen species, which can damage proteins, DNA, lipid function, trigger cell death signaling pathways, and raise MDA levels in tissues.

Modulation of important biochemical pathways related to oxidative stress and inflammation may be responsible for the protective benefits seen in this study after injection of the conditioned medium obtained from bone-marrow stem cells. In particular, BMSCs-CM is anticipated to decrease the NF-κB signaling pathway, which is crucially linked to pro-inflammatory responses and tissue damage, while simultaneously promoting activation of the Nrf2 signaling pathway, a master regulatory axis of antioxidant gene expression. In experimental models of organ injury, for example, extracellular vesicles produced from mesenchymal stem cells have been demonstrated to suppress NF-κB-mediated inflammation and activate the Nrf2/HO-1 axis. The involvement of these molecular pathways offers a tenable mechanistic explanation in the context of the current findings, namely the significant decrease in malondialdehyde levels, increase in catalase activity, and near-normal histological appearance of both the salivary gland and kidney following BMSCs-CM treatment. Additionally, BMSCs-CM provides a cell-free substitute for other antioxidant strategies or stem cell-derived therapies that may get around a number of restrictions related to cell transplantation (such as immune rejection, oncogenic potential, and logistical complexity) while producing similar modulatory effects on oxidative/inflammatory mediators [59].

Boosting of natural antioxidant defenses and reduction of pro-inflammatory signaling are two important pathways that may explain the protective impact of BMSC-derived conditioned medium seen in this work. According to earlier research, MSC-CM has a wide variety of antioxidant enzymes and redox-regulating proteins (including SOD1 and the PRDX family) and can repair oxidative damage by up-regulating these enzymes and lowering ROS levels [60]. Furthermore, Jiao et al. stated that MSC-derived secretomes regulate the NF-κB pathway, reducing inflammatory mediator production and reducing oxidative-inflammatory cross-talk in damaged tissues [61]. When considered collectively, these results support the theory that the CM in our model may have reduced oxidative stress and organ damage by boosting catalase and superoxide dismutase activity while simultaneously suppressing NF-κB-mediated pro-inflammatory reactions.

In addition to oxidative stress, the release of proinflammatory cytokine has also been implicated in the pathogenesis of Amiodarone toxicity. Oxidative environment leads to activation of nuclear factor kappa B and increased production of cytokines, including tumor necrosis factor-α (TNF-α) [62]. TNF-α is a cytokine that released by lymphocytes and macrophages, plays a crucial role in inflammation, apoptosis, cell differentiation, and acquired and innate immunity [63]. The increase in the synthesis of proinflammatory cytokines expand the tissue damage by raising the amount of ROS [64]. Our study's results showed that the Amiodarone group's blood TNF-α and IL-6 levels considerably elevated when compared to the control and CM group, suggesting increased levels of damage caused by Amiodarone and anti-inflammatory properties of BMSC-CM. In agreement with our findings Lo Sicco and Jin QH et al. [65, 66] stated that MSC-CM possess anti-inflammatory properties and can reduce levels of pro-inflammatory cytokines such as IL-6 and TNF-α. Consistent with our findings, Amiodarone has been shown to raise IL-6 [67] and TNF-α [68] in rat kidney tissue. Also, Mohammed WI et al., Fattiny, S.Z.A et al. and Dagel T et al. [69–71] recorded significant increase in urea, creatinine, TNF-α and MDA levels in rats received Amiodarone induced damage in rats. Previous studies have illustrated that increased levels of proinflammatory cytokines in different tissues due to Amiodarone also developed sever histopathological damage [71, 72]. According to reports, MSCs-CM encourage macrophages to take on an M2 phenotype that is regulatory in nature. Pro-inflammatory cytokine production was markedly decreased in this phenotype, while IL-10 production was elevated. hence having immunosuppressive and anti-inflammatory effects [73]. These findings align with the experimental findings of Tuncay Dagel et al. [71], who discovered that Amiodarone raised creatinine levels and decreased CAT levels in rat kidney tissues while raising MDA, TNF-α, and IL-6 values.

Histological analysis of the salivary gland of Amiodarone group revealed cytoplasmic vacuolation of the acinar cells, as well as degeneration and luminal shedding of the striated duct lining epithelium. Additionally, interacinar blood capillary hemorrhage was seen. Significant acini and duct degeneration, and thickening and enlargement of interstitial gaps are also observed. These symptoms align with the conclusions of Alev-Tuzuner et al. [4], who used Amiodarone to induce toxicity in parotid glandes of experimental rats. Amiodarone's oxidant effects increase tissues oxidative stress that leads to the increased production of reactive oxygen species, which damage the membrane lipids, proteins, and DNA [74]. Oxidative stress in the salivary gland causes inflammation, secretory dysfunction and damage to cellular structures, which could result in long-term functional impairment [75]. Emerging evidence suggests that oxidative stress affects salivary gland function by promoting lipid peroxidation, disrupting enzymatic activities, and altering levels of essential biomolecules involved in cellular defense and signaling. Zalewska et al., Stevens and Lowe, 2000 [24, 76] postulated that the cytoplasm's vacuolation might result from mitochondrial injury that causes the cell to malfunction metabolically, allowing sodium ions to enter the cell. The big macromolecules in the injured cell are broken down by their osmotic action, which results in cytoplasmic vacuolation. These results tended to decline in CM groups; compared to the Amiodarone group, there were clearer lobular structures with fine intra-lobular gaps, and a greater number of acini and ducts with normal nuclei and fewer vacuoles, these findings were apparent signs of improvement and healing suggesting the anti-apoptotic, antioxidant and anti-inflammatory role of BMSCs-CM. The results of the present study are in line with Zakaria et al. [77] who state that BMSCs-exosomes restore the architecture structure of parotid salivary gland after its intravenous injection in albino rats subjected to cisplatin induced damage through paracrine mediators including growth factors and chemokines that have chemoprotective effects.

Histological analysis was also performed on the kidney tissues taken from the experimental groups. The Amiodarone group's histopathological data revealed glomerular injury, interstitial hemorrhage and renal tubular vacuolar degeneration and dilatation, all signs of acute kidney injury. Our results were confirmed by Sakr and El-Gamal 2016 [49] who reported that Amiodarone treated rats showed renal tubules appeared with wide lumen and the tubular epithelia were degenerated and their lining cells exhibited cytoplasmic vacuolation and pyknotic nuclei as well as the glomeruli were atrophied with dilatation in the subcapsular space and congested renal veins. In the same context, Fattiny and Al-Amin 2019 [70] discovered that the amiodarone group rats increased renal tubular vacuolization and necrosis. Amiodarone was found to be the cause of severe tubular degeneration. According to our findings, the histopathological changes of renal tissue were markedly decreased in groups that received BMSCs-CM treatment; this is in agreement with Salem et al. who said that rats given MSCs-CM appeared to have improved kidney morphology, as evidenced by H&E-stained sections that revealed a reduction in cell vacuolation. Additionally, the study by Abouelkheir et al. [51] showed that MSCs and their CM improved kidney functioning, reduced apoptosis, encouraged proliferation, and lessened pathological alterations. Conversely, in rats with acute kidney injury caused by cisplatin, Gheisari et al. [78] discovered that MSCs-CM were unable to prevent kidney failure based on serum urea and creatinine levels as well as histopathologic investigations.

Masson's trichrome stain is commonly used to assess collagen deposition and fibrosis in tissues; it is not directly associated with damage induced by Amiodarone toxicity. Instead, Amiodarone induced salivary gland damage as a result of oxidative stress, inflammation, and changes in collagen content. Statistically, our results demonstrated collagen fibers were excessive, looser and less organized in the Amiodarone group's reflecting fibrosis and degeneration. while CM treated group exhibiting moderate collagen deposition and reduced fibrotic changes suggesting protective and anti-apoptotic effects of the CM treatment. The present findings are in agreement with Sharaf El-Din and Abd Allah (2016) & Mahdy (2014) [79, 80] who found that amiodarone toxicity increased the collagen deposition and fibrosis. El Bana and Shawky (2019) [81] also found that Amiodarone increases the collagen deposition in lung and regression occurred by BMSCs therapy. Zaglool et al. [82] stated that Amiodarone induce fibrosing lung disease in albino rat and cord blood MSC therapy proved definite Improvement. This was supported by Kim et al., 2016, Chen et al., Mehrabani et al. and Khajeha hmadi et al. [42, 83–85] who found that MSCs-CM had anti-inflammatory and healing effect in rearrangement of fibrosis in different organs. The primary mechanism stimulating collagen expression with excessive collagen production was the lipid peroxidation process [86]. Amiodarone was found to upregulate angiotensinogen messenger RNA and angiotensin II that shown to promote fibrosis through stimulation of transforming growth factor-b1 [87].

In the current study, caspase 3 antibodies demonstrated a strong positive reaction to activated caspase 3 in the cytoplasm of Amiodarone group indicating extensive apoptosis, while control and conditioned media groups displayed mild immunoreactivity against caspase 3 antibody [88]. Amiodarone induces apoptosis in salivary gland and kidney through caspase activation, a key enzyme in the apoptotic pathway, leading to cell death. This can lead to salivary gland and kidney dysfunction. The ability of CM to neutralize reactive oxygen species, functioning as a strong scavenger of free radicals, makes it as a promising candidate for therapeutic use. Furthermore, the decrease in the expression of Caspase 3 antigen in CM group, in comparison to Amiodarone group, indicates a decrease in proapoptotic activity, which is a positive indication of the protective impact of BMSCs-CM. Kapatou et al. [89] found that amiodarone increases apoptosis by decreasing the expression of anti-apoptotic proteins and by increasing expression of caspase 8. Farouk et al. [90], also stated that Amiodarone increase the expression caspase 3 protein, indicating enhancement of apoptosis.

## Conclusions

Based on the data and the methodology employed, the present study detected functional and histopathological toxic effects of Amiodarone that can harm the salivary glands and kidney. Because BMSCs-CM has anti-inflammatory and anti-oxidative qualities and restores oxidant/antioxidant equilibrium, rats treated with it recover faster and are better equipped to resist Amiodarone-induced injury to their salivary glands and kidneys. These findings suggest that BMSCs-CM could represent a promising cell-free therapeutic strategy to minimize drug-induced salivary and renal toxicity in clinical settings.

### Limitations

One of the study's limitations is that only adult male rats were utilized; females were not included to prevent hormonal fluctuation, which restricts the data' generalization to both sexes. Furthermore, only one treatment period was assessed, making it unable to assess the long-term or preventive effects of BMSCs-CM. Morphometric investigations were not carried out, and the histological evaluation was mostly qualitative. Future research using these quantitative and molecular methods would confirm the reported histological and biochemical results in a more thorough and objective manner.

### Recommendations

The findings of this research indicate that BMSCs-CM media has a significant protective effect against toxicity in the salivary glands and kidneys after Amiodarone exposure. Future research should involve both male and female participants to evaluate any sex-related differences in treatment response. Additionally, it is advised that forthcoming studies explore the preventive effects of BMSCs-CM. Further studies should test different doses and administration routes of BMSCs-CM to optimize its therapeutic efficacy.

## Data Availability

All data generated during this study are included within this article.

## Abbreviations

BMSCs: Bone marrow mesenchymal stem cells
BMSCs-CM: Bone marrow mesenchymal stem cells conditioned media
CAT: Catalase
CM: Conditioned media
H&E: Hematoxylin and Eosin
IL-6: Interlukin 6
IL-10: Interlukin 10
L-DMEM: Low glucose Dulbecco’s Modified Eagle’s Medium
MSCs: Mesenchymal stem cells
MSCs-CM: Mesenchymal stem cells conditioned media
MDA: Malondialdehyde
PBS: Phosphate buffer saline
PDGF: Platelet derived growth factor
SMSGs: Submandibular salivary glands
